# Structure-guided computational insecticide discovery targeting *β*-N-acetyl-D-hexosaminidase of *Ostrinia furnacalis*

**DOI:** 10.1080/07391102.2023.2264394

**Published:** 2023-10-09

**Authors:** Adeena Tahir, Abdul Rauf Siddiqi, Arooma Maryam, Sundeep Chaitanya Vedithi, Tom L. Blundell

**Affiliations:** a Department of Biosciences, COMSATS University Islamabad (CUI), Islamabad, Pakistan; b Department of Biochemistry, University of Cambridge, Cambridge, UK; c Department of Medicine, University of Cambridge, Heart and Lung Research Institute (HLRI), Cambridge, United Kingdom

**Keywords:** β-N-acetyl-D-hexosaminidase, *Ostrinia furnacalis*, virtual screening, MD simulations, reference docking, insecticides

## Abstract

*Ostrinia furnacalis* is a species of moth in the Crambidae family that is harmful to maize and other corn crops in Southeast Asia and the Western Pacific regions. *Ostrinia furnacalis* causes devastating losses to economically important corn fields. The β-N-acetyl-D-hexosaminidase is an essential enzyme in *O. furnacalis* and its substrate binding +1 active site is different from that of the plants and humans β-N-acetyl-D-hexosaminidases. To develop environment-friendly insecticides against OfHex1, we conducted structure-guided computational insecticide discovery to identify potential inhibitors that can bind the active site and inhibit the substrate binding and activity of the enzyme. We adopted a three-pronged strategy to conduct virtual screening using Glide and virtual screening workflow (VSW) in Schrödinger Suite-2022-3, against crystal structures of OfHex1 (PDB Id:3NSN), its homologue in humans (PDB Id: 1NP0) and Alphafold model of β-N-acetyl-D-hexosaminidase from *Trichogramma pretiosum*, an egg parasitoid that protects the crops from *O. furnacalis.* A library of 20,313 commercially available and “insecticide-like” compounds was extracted from published literature. LigPrep enabled 44,943 ready-to-dock conformers generation. Glide docking revealed 18 OfHex1-specific hits that were absent in human and *T. pretiosum* screens. Reference docking was conducted using inhibitors/natural ligands in the crystal structures and hits with better docking scores than the reference were selected for MD simulations using Desmond to understand the stability of hit-target interactions. We noted five compounds that bound to OfHex1 TMX active-site based on their docking scores, consistent binding as noted by MD simulations and their insecticide/pesticide likeliness as noted by the Comprehensive Pesticide Likeness Analysis.

Communicated by Ramaswamy H. Sarma

## 1. Introduction

According to the Food and Agriculture Organization (FAO) of the United Nations, worldwide loss of agricultural yield due to pests and diseases has been predicted to be 40% (Secretariat et al., 2021). Traditional plant protection methods, including the use of conventional insecticides, have played a significant role in boosting agricultural productivity and remain the major prominent tool in modern agriculture and pest management. (Speck-Planche et al., 2012). However, as with any alternative, the insecticides also come with serious toxicological, environmental, hazards and insect resistance problems due to their extensive non-selective utilization (Perry et al., 2011; Harrison & Bonning, 2010).

Amid these challenges, it is noteworthy that few of the current generation of chemical insecticides act primarily on evolutionary conserved protein targets among all insect species, as well as mammals. Chemical insecticides that act on highly conserved protein targets across insects and mammals not only affect harmful insects but also cause collateral damage to beneficial insects and mammals (Perry et al., 2011). Target conservation maximizes the challenge of discovering selective compounds exhibiting lower associated risks. Growing awareness against the ecological problems related to indiscriminate use of insecticides justifies the necessity of research to discover target pest protein-specific insecticide. In light of discovering targeted insecticides, researchers have explored innovative approaches to combat destructive pests like *Ostrinia furnacalis* (*O. furnacalis*), which inflicts substantial damage on maize *(Zea mays L.)* fields globally (Guo et al., 2019).

*Ostrinia furnacalis* (*O. furnacalis*) causes devastating losses to economically important corn fields in China, the Philippines, Japan, Korea, Thailand, Indonesia, Malaysia, and other countries. The life cycle of *O. furnacalis* is driven by latitude and climate, resulting in varying generations on a scale from one to seven per year (All China Corn Borer Research Group, 1988; Guo et al., 2022). One of the promising avenues lies in the development of targeted insecticides that disrupt specific processes critical to pest survival. Enzyme inhibition has emerged as a powerful tool, β-N-acetyl-D-glucosamine (Hex) enzymes are crucial for hydrolyzing terminal GlcNAc or GalNAc from the diverse saccharides’ non-reducing ends. Recently, insecticide inhibitors with specific activity against *O. furnacalis*’s chitinolytic β-N-acetyl-D-hexosaminidases (OfHex1) have been discovered. In this study, authors have investigated the specificity of naphthalimide against humans, insects and bacterial chitinolytic β-N-acetyl-D-hexosaminidase (T. Liu et al., 2014). Potential inhibitors of OfHex1 have also been reported by Liu et al. (2012). These findings gain additional significance as they correlate with the information provided by the Carbohydrate-Active enZYmes (CAZy) database (http://www.cazy.org/).The CAZy database provides insights into catalytic and carbohydrate-binding module (or functional domain) families of enzymes, including glycosyl hydrolases (GH), which have been categorized into family groups such as GH3, GH20, and GH84 (Cantarel et al., 2009). Among these, OfHex1, a glycosyl hydrolase belonging to the GH20 family, emerges as a crucial enzyme for the breakdown of insect chitin. It has been recognized as a potential enzyme target for the development of environmentally friendly insecticides because of its role in disrupting fundamental insect processes, including growth, pupation, molting, and eclosion, ultimately leading to insect mortality when inhibited (Muthukrishnan et al., 2020; Zhang et al., 2022). Since humans, plants and vertebrates do not possess or process chitin, these enzymes are considered to be potentially specific and reliable targets for identifying and designing green pesticides (Dong et al., 2021). Chitinolytic β-N-acetyl-D-hexosaminidases differ from chitinases, by exhibiting specialization in insect chitin catabolism while remaining absent in viruses, plants, and animals(J. Liu et al., 2012). This unique specificity underscores their potential as targeted and effective agents against pests like *Ostrinia furnacalis* which inflicts substantial damage on economically significant crops.

So far, many OfHex1 inhibitors have been reported and among these TMG-chitotriomycin, produced by *Streptomyces anulatus*, exhibited no measurable inhibition of human β-N-acetyl-D-hexosaminidases and was shown to be a highly potent and effective OfHex1 inhibitor (Ki = 0.065 μM) (Usuki et al., 2008). Building upon the quest for selective and potent insecticide leads against OfHex1, the search for effective inhibitors continues due to challenges related to synthesis and drug-like properties.

To address innovative solutions for pest control and to create a sustainable balance between agricultural productivity and ecological health, targeted enzyme inhibition should be combined with strategic utilization of biological controls Presently, *Trichogramma* species are broadly used biological antagonists, mainly against Lepidopteran insects during the egg stage in their life cycle. *Trichogramma pretiosum (T. pretiosum*), as well as many other species, have been extensively investigated as biocontrol agents in many crops like maize, cotton, rice and sugarcane, in various vegetables, forestry and for insects in stored food. Ensuring the protection of these egg parasitoids alongside targeted insecticide applications is critical to control the damage caused by harmful pests (El-Wakeil et al., 2013)^.^

Responding to these challenges, computational insecticide designing techniques are now introduced in agrochemical industries, which act as a promising solution to discover and develop novel and target-specific insecticides (Speck-Planche et al., 2012). In this work, we have carried out virtual screening of existing insecticides, including diverse chemical scaffolds (extracted from targeted libraries aiding insecticide discovery), to discover OfHex1-specific inhibitors. All the compounds were subjected to a VLS protocol that involved multistep docking and molecular dynamics simulations against crystal structure of OfHex1 (PDB Id: 3NSN) (T. Liu et al., 2011), human β-N-acetyl-D-hexosaminidase (PDB Id: 1NP0) (Mark et al., 2003), and a homology model of *T. pretiosum* Hex, to identify the unique and specific compounds against OfHex1 (Jacobson et al., 2004).

We conducted high throughput virtual screening using the existing insecticidal compounds and chemical scaffolds to maximize search space for the identification of specific OfHex1 inhibitors and used MD simulations to investigate the stability of various ligand bound complexes, which might be helpful to analyze the interactions between Hex and its potential inhibitors and also provide promising insights into the future development of novel environmentally friendly insecticides.

## 2. Materials and methods

### 2.1. Target identification and comparative modelling

In the current study, β-N-acetyl-D-hexosaminidase from *O. furnacalis* has been used as a potential target for insecticide. To explore target and organism-specific insecticides, β-N-acetyl-D-hexosaminidases from three different organisms were selected: *O. furnacalis* (pest), *Homo sapiens* (mammal), and *T. pretiosum* (egg parasitoid). Co-crystalized structure of OfHex1 complexed with TMG-chitotriomycin (PDB Id: 3NSN) (T. Liu et al., 2011) and human β-N-acetyl-D-hexosaminidase complexed with intermediate analogue NAG-thiazoline (PDB-Id: 1NP0) (Mark et al., 2003) were retrieved from Protein Data Bank (PDB). A comparative sequence and structural analysis of OfHex1 and human β-N-acetyl-D-hexosaminidase reveal a sequence identity of 40.11% and a whole atom RMSD of 1.210.

For *T. pretiosum,* (Sousa et al., 2017) a computational model was built using Alphafold-2, and model quality was estimated using predicted least distance difference test (pLDDT) and Molprobity (Williams et al., 2018). The pLDDT score was 86.750, Molprobity score was 1.61 at the 92^nd^ percentile (*N* = 27675, 0 Å-99Å) and the clash score was 1.75 at the 99^th^ percentile (*N* = 1784, all resolutions). The RMSD and sequence identity of the model with its template (PDB Id: 5Y1B - OfHex1 complexed with a berberine derivative) (Duan et al., 2018) was 0.750 Å and 36.41% respectively.

All these protein targets were subjected to Protein Preparation Wizard in the Maestro interface accessible through Schrödinger Small molecule drug discovery suite 2022-04 (www.schrodinger.com). All unnecessary heteroatoms including waters (that are more than 5 Å) were removed. Missing sidechain atoms and hydrogen atoms were added using PRIME, and restrained minimization was performed to remove steric clashes using the OPLS4 force field.

### 2.2. Ligand retrieval and preparation

A virtual library of a collection of 20,313 commercially available and “insecticide-like” compounds were obtained from different databases as shown in Table 1. Ligprep (Schrödinger, LLC, New York, NY, 2021) application was used to generate all possible ionization states and stereoisomers using the OPLS4 force field. For each compound, a maximum of 32 conformations were generated. Subsequent to ligand preparation, a total of 44,943 ready-to-dock insecticidal molecules were selected for structure-based virtual screening.

**Table 1.** Sources of chemical databases along with their number of inputs and generated conformers. The source of each database is also enlisted.

Ligand database	Category	No. of insecticides/ core chemical scaffolds	No. of conformers generated by Ligprep	Source
ChemFACES	Insecticides Compounds Library	90	521	(Rona et al., 2022)
LifeChemicals	Developed Insecticides Library	1831	3097	(Odhar et al., 2021)
InsectiPAD	Fully Developed Insecticides	512	2406	(Jia et al., 2019)
Literature & PubChem*	Insecticides	81	122	(Rona et al., 2022)
Otava Chemicals	Insecticides Like Library	1984	3617	(Is et al., 2020)
Otava nACHr	Predicted insecticides Library targeting nicotinic acetylcholine receptor	499	857	(Is et al., 2020)
EU Pesticide Database	Approved Insecticide Compounds only	330	438	(Marchand, 2018)
Enamine Insecticides Clusters	Clusters of S-aliphatic and Hal substituted insecticides	13,042	31,272	(Tenhunen et al., 2021)
ChemDIV	Insecticide Like Compounds	1,944	2,613	(Chen et al., 2022)
Total		20,313	44,943	

* A total of 31 compounds were extracted from literature (Banik et al., 2020) and 50 insecticidal compounds were extracted from PubChem database.

### 2.3. Molecular docking protocol

For molecular docking, co-crystallized ligands were redocked first to predict the probable binding modes of the compounds. For reference docking, initially, receptor grids were generated using three-dimensional (3D) coordinates of various atoms in TMG-chitotriomycin and NAG-thiazoline with OfHex1 (PDB Id: 3NSN) and human β-N-acetyl-D-hexosaminidase (PDB Id: 1NP0) respectively. Dockings were repeated with grids generated for each of the heteroatoms in the complex until the closest conformation to the native ligand was achieved. For the generation of grid boxes, the active sites were surrounded within 10 Å length of the centroid atom selected manually and identified to have the closed pose with the reference ligand (recognized by lowest RMSD) (Tahir et al., 2018). Arpeggio was used to understand and calculate the interactions between the template (PDB Id: 5Y1B) and its ligand 8KL (Berberine Derivative) and the *T. pretiosum model* with its template’s ligand (Jubb et al., 2017).

**Figure 1.**
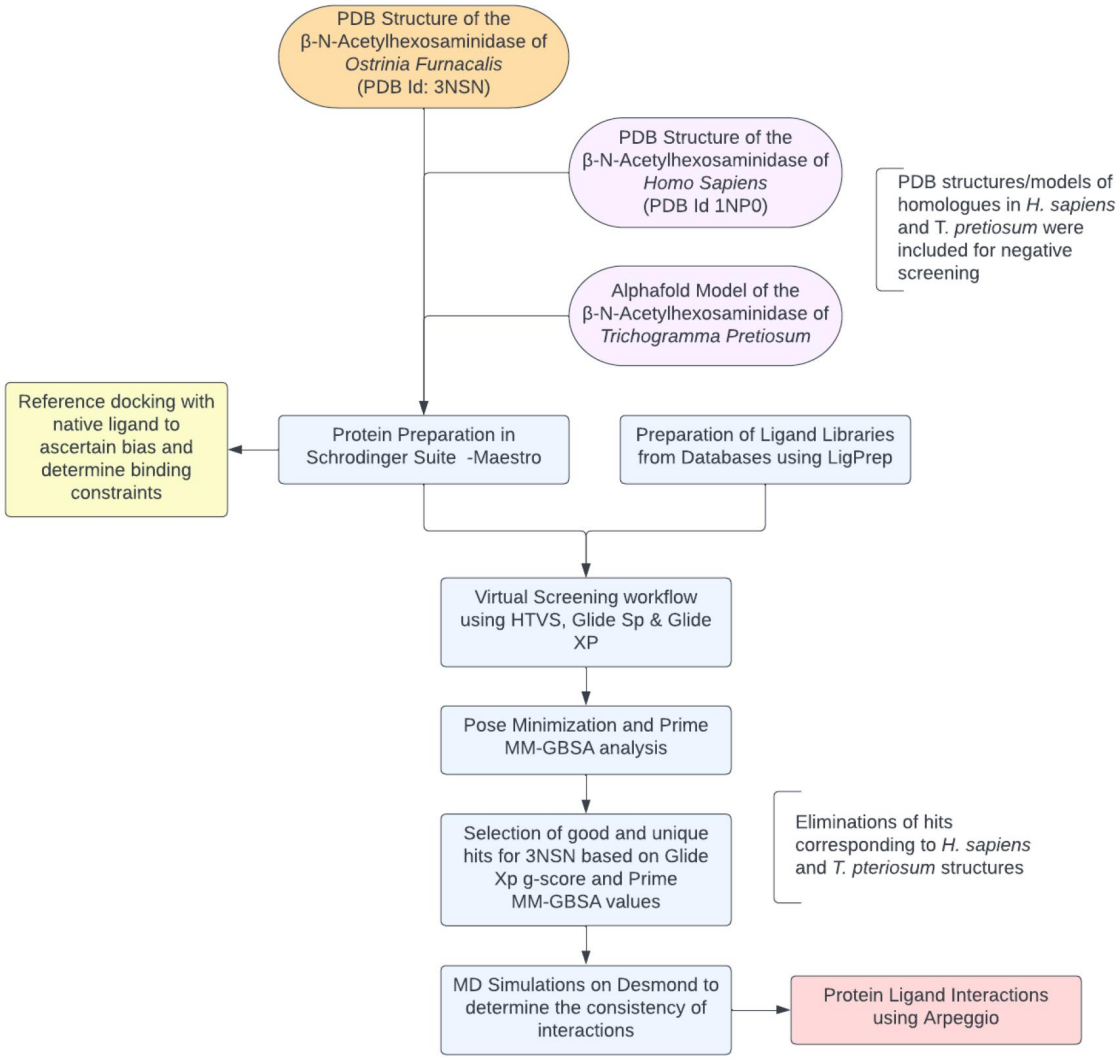
The above methodology was adopted which included multistep virtual screening workflow along with MM-GBSA calculations and MD simulations to prioritize small molecules that can potentially inhibit OfHex1 activity in *O.* *furnacalis.*

Later, the prepared ligand dataset was screened against the binding pockets of the aforementioned structures or models using Glide extra precision (XP) docking algorithm (Friesner et al., 2004). For the top 3 XP docked poses, Prime MM-GBSA energy changes were calculated. During docking, softening the potential for the nonpolar parts of the receptor was applied by adjusting the scaling factor of van der Waals radii to 0.80 with a cut-off value of 0.15 along with other default parameters. Among all the plausible conformers of docked compounds, energy minimization of only the top three poses of each compound was done. Eventually, the best pose was selected based on the highest Glide score, residue interactions, best binding mode and MM-GBSA score (Pattar et al., 2020). The more promising compounds were further subjected to molecular dynamics (MD) simulations and the binding free energies were calculated by using the molecular mechanics-generalized Born surface area (MM/GBSA) methodology (Bowers et al., 2006) (Godschalk et al., 2013). The general process of filtering the insecticide compounds/core chemical scaffolds library by multistep approaches used in this study are presented in Figure 1.

### 2.4. Molecular dynamics (MD) simulations

After selection of top five docked complexes based on the Glide XP G-scores, MD simulations were carried out to identify the stability of interactions between the selected compounds and OfHex1 using the Desmond Schrödinger LLC package (Bowers et al., 2006). The System Builder module helped prepare the solvent system for simulation. A cutoff radius of 10.0 Å in Coulomb interactions, and orthorhombic periodic box boundaries were set away from the protein atoms at 10 Å making sure the entire complex was enclosed by the solvent molecule. The water molecules were described using the TIP3P model. The molecular systems were neutralized by adding Na + ions. The Desmond protocol includes a gentle restrained minimization and relaxation of the solvated built system using OPLS4 force field parameters (Lu et al., 2021).

Default quick relaxation protocol was used to refine and optimize energy of all the five complexes. In brief, steepest descent minimization was performed in two rounds, with a maximum of 2000 steps, in the presence and absence of restraints (force constant of 50 kcal/mol/Å on all solute atoms). Afterwards, four quick MD simulations runs were performed: **(1)** 12ps (picoseconds) MD simulation at (10 K temperature) in the Berendsen NVT (constant number of atoms N, volume V and temperature T) ensemble restraining non-hydrogen solute atom (force constant of 50 kcal/mol/Å); **(2)** 12ps (picoseconds) MD simulation at (10 K temperature) in the Berendsen NPT (constant number of particles, pressure, and temperature) ensemble; **(3)** 12ps (picoseconds) MD simulation at (temperature raised to 300 K) in the Berendsen NPT ensemble with the same restraints as in **(1)** and **(2)**; **(4)** 24ps (picoseconds) MD simulation at (300 K temperature) in the Berendsen NPT ensemble without restraints.

During MD simulations, the Root Mean Square Deviations (RMSD) from the preliminary structure and the root mean square fluctuations (RMSF) of all atoms were calculated. Atomic spacing and different structural conformations significant for binding the ligand were also considered (Lindin et al., 2014). For the simulation interaction analysis, MD Simulations trajectories of the top 5 complexes with their respective frames were saved and the simulation interaction diagrams analyzed using event analysis module in Desmond (Bowers et al., 2006). Prime MM-GBSA analysis was carried to estimate ligand strain energies and the ligand binding free energies for docked molecules over the 40 ns period (Pattar et al., 2020).

## 3. Results and discussion

**Figure 2.**
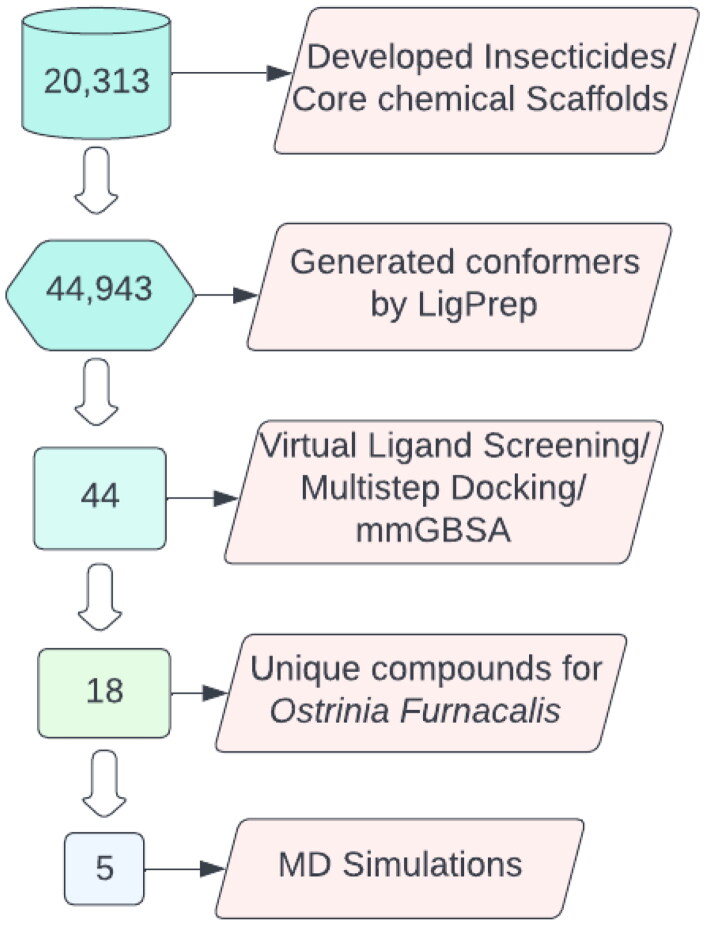
Flowchart of compounds filtered by virtual ligand screening/multi-step molecular docking and MD simulation. After extracting the crystal structures of OfHex1 of *O. furnacalis* and *H. sapiens* and building the Alphafold model of *T. Pretiosum*, the developed insecticide/core chemical scaffold library was screened using glide molecular docking workflow. Top-ranked compounds that uniquely bind to OfHex1 and have scores better than reference docking were selected. Molecular dynamics simulations and MM-GBSA calculations were carried out to determine the consistency of molecular interactions.

In the past, agrochemical discovery has been done through *in vivo* and *in vitro* high throughput screening, but both of these random screening approaches involve comprehensive data of compounds and utilization of a significant number of resources. Nevertheless, generation of only a few hits that can progress to potential leads and fully developed insecticide compounds is usually achieved (L. Wang et al., 2010; Lavecchia & Giovanni, 2013). In this context, *in silico* screening approaches have gained acclaim and acceptance. Computational insecticide designing techniques are now introduced in agrochemical industries to invent novel pesticides on the basis of biological targets (Speck-Planche et al., 2012). Virtual Ligand Screening (VLS) represents a crucial step in the early pesticide designing process for the discovery of compounds based on pesticide-likeness criteria (Martinez-Mayorga et al., 2020). VLS can be an efficient approach to find potent inhibitors and for identifying novel ligands against unexplored putative insecticide targets for which structural data are available in publicly available data repositories (Lavecchia & Giovanni, 2013). In this study, virtual screening approach is used to produce a targeted and active subset of compounds from the aforementioned chemical library. The top ranked hits filtered from the library of insecticide compounds/core chemical scaffolds by multistep approaches are presented in Figure 2. After selecting insecticides/core chemical scaffolds from multiple databases, we employed a virtual ligand screening protocol to filter the OfHex1-specific insecticide compounds. Top scored hits that only show binding with *O. furnacalis* but not with the homologues in *H. sapiens* and *T. pretiosum*, with favorable XP docked conformations were selected, and then the MD simulations and ‘Molecular Mechanics with Generalized Born and Surface Area’ (MM-GBSA) binding free energy calculations for these selected docking complexes were performed.

Information on all the compounds docked with *O. furnacalis* β-N-acetyl-D-hexosaminidase, *T. pretiosum* β-N-acetyl-D-hexosaminidase homology model and *H. sapiens* β-N-acetyl-D-hexosaminidase (1np0) is presented in Supplementary Data 1 (Tables 1–3 respectively). Details of the virtual screening approach, MD simulations, and the binding free energy calculations of the docked complexes specific to *O. furnacalis* are presented below.

**Table 2.** Calculated lead-like properties, MMGBSA bind energy, and docking scores of 18 virtual screening hits.

No.	**PubChem ID** ^†^	Formula	Chemical Structure	**HBA** *	**HBD** *	Mw	Glide g-score	Glide energy	MM-GBSA dG Bind
1	95203364	C_15_H_17_F_4_N_3_O	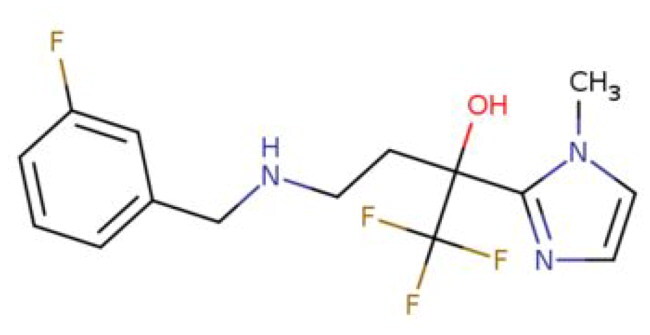	3	2	331.312	−13.68	−46.251	−68.81
2	19591105	C_16_H_20_Cl_2_N_2_O_2_	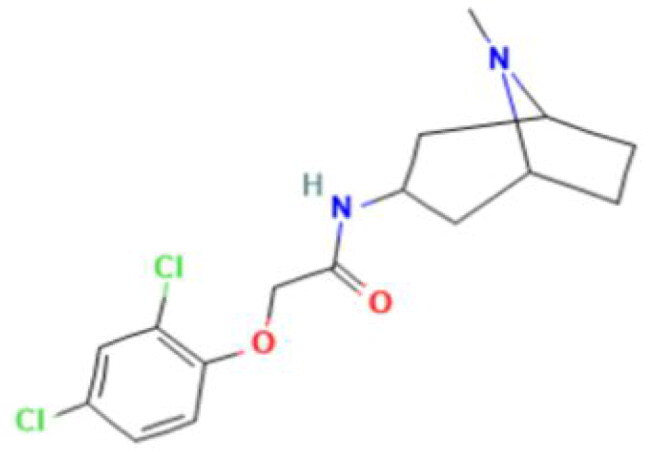	3	1	343.252	−12.96	−42.15	−41.55
3	48957080	C_16_H_21_F_4_N_3_O	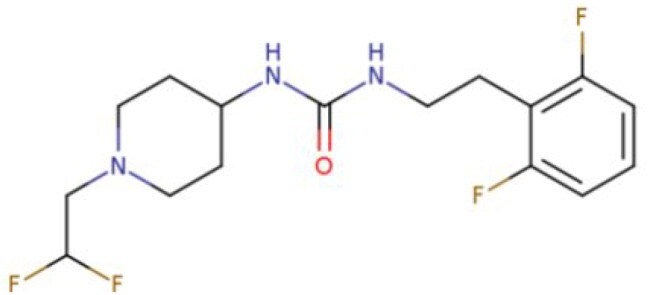	2	2	347.355	−13.921	−37.643	−29.73
4	56783035	C_16_H_20_F_4_N_2_O_2_	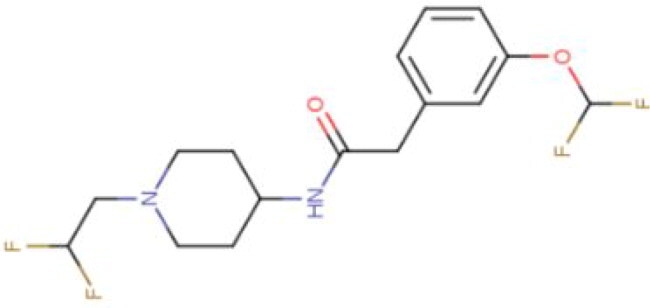	3	1	348.34	−14.834	−46.969	−24.1
5	53498488^†^	C1_4_H_27_N_3_O_4_S	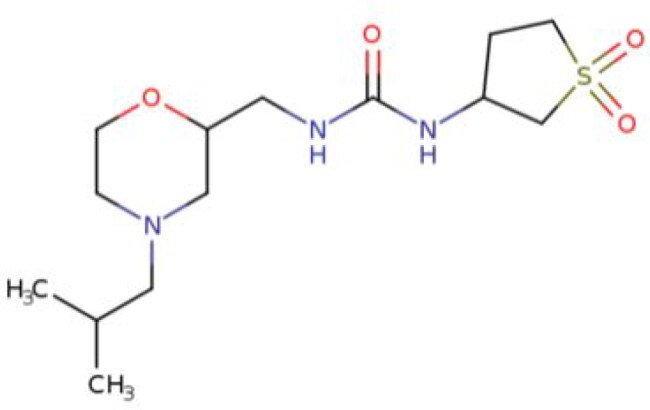	5	2	333.445	−13.134	−43.849	−38.19
6	53498488^†^	C1_4_H_27_N_3_O_4_S	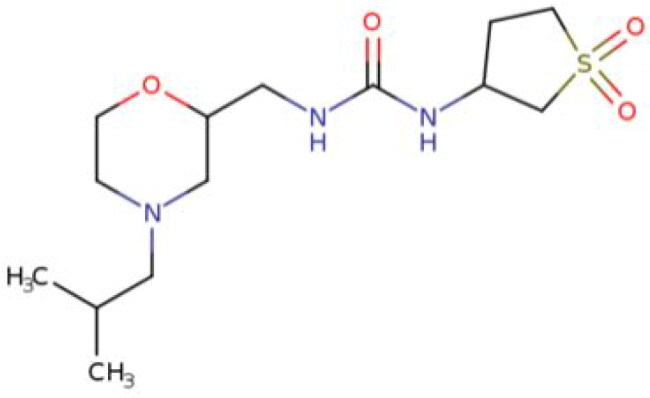	5	2	333.445	−13.21	−37.676	−17.98
7	71867970^†^	C_17_H3_4_N_4_OS	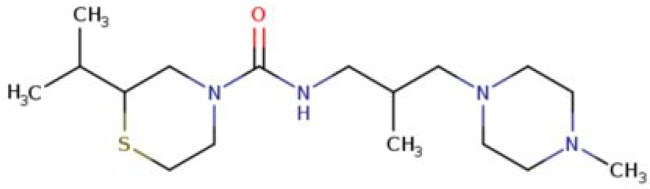	3	1	342.542	−14.028	−48.561	−53.21
8	71867970^†^	C_17_H_34_N_4_OS	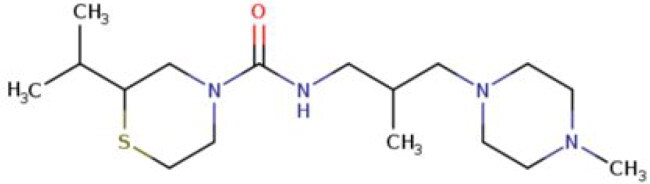	3	1	342.542	−13.416	−43.691	−38.72
9	75432522^†^	C_15_H_21_F_4_N_3_	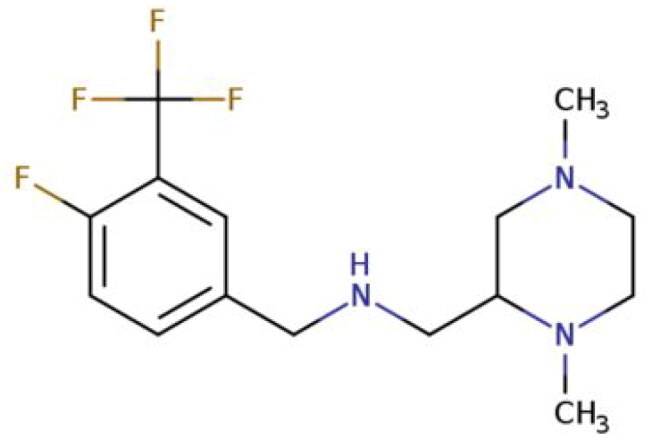	5	1	319.345	−15.936	−49.646	−43.73
10	75432522^†^	C_15_H_21_F_4_N_3_	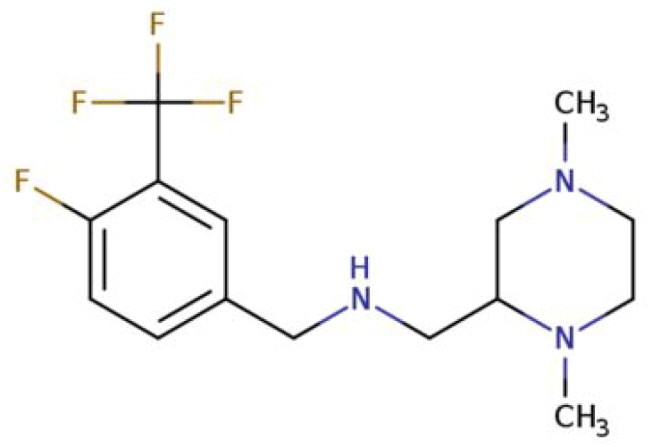	5	1	319.345	−13.86	′44.739	−50.51
11	797993	C_14_H_20_N_2_O	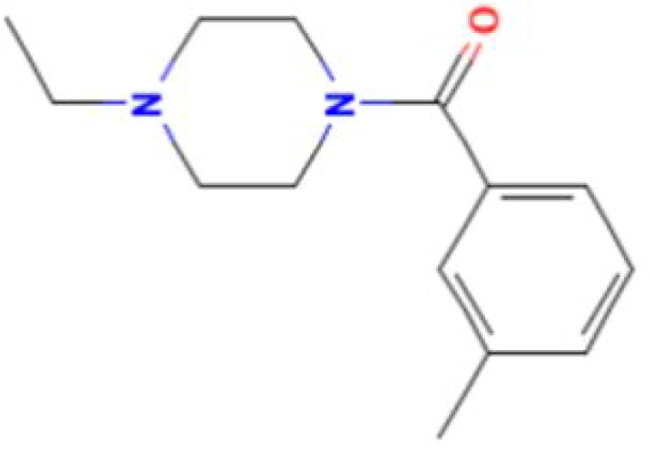	2	0	232.325	−13.206	−31.382	−32.59
12	97230286	C_16_H_22_F_4_N_2_	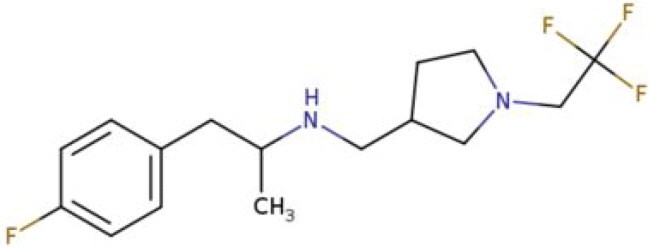	2	1	318.357	−14.549	−44.002	−44.31
13	56788228	C_16_H_21_F_4_N_3_O	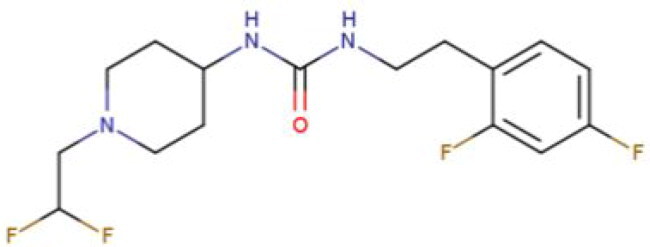	2	2	347.355	−14.495	−43.43	−28.25
14	75382816	C_15_H_18_F_6_N_2_	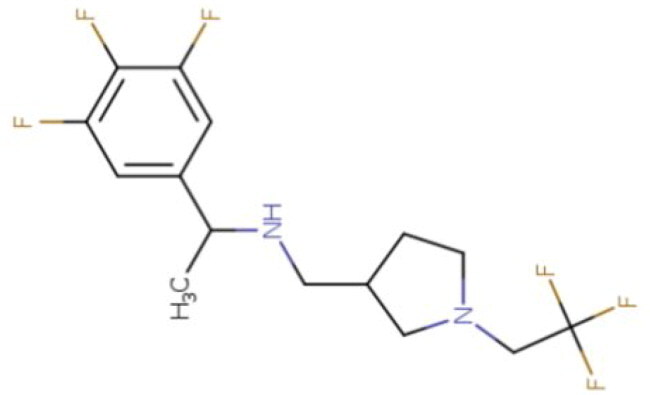	2	1	340.311	−14.219	−48.449	−45.56
15	25271794	C_13_H_22_N_5_O_2_	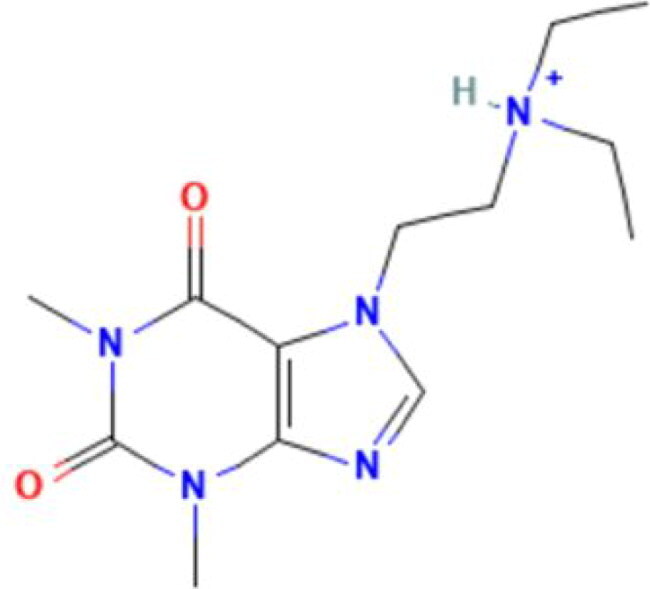	3	1	279.341	−13.106	−44.673	−52.03
16	75431867	C_15_H_21_ClF_3_N_3_	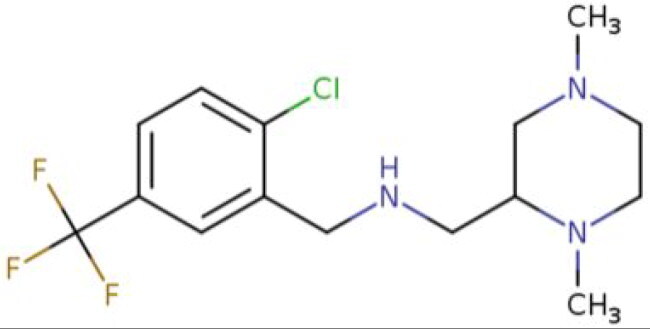	5	1	335.799	−15.876	−50.361	−44.74
17	53590748	C_15_H_19_F_4_N_3_O	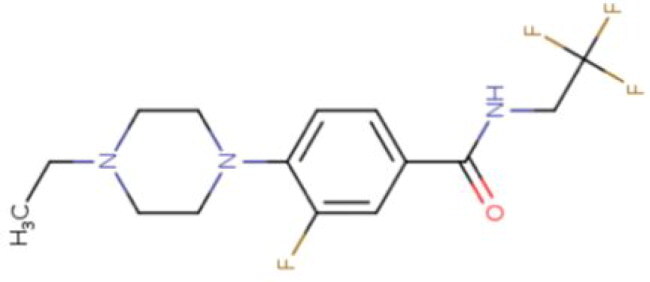	3	1	333.328	−13.416	−41.847	−28.79
18	72007806	C_15_H_17_F_4_N_3_O_2_	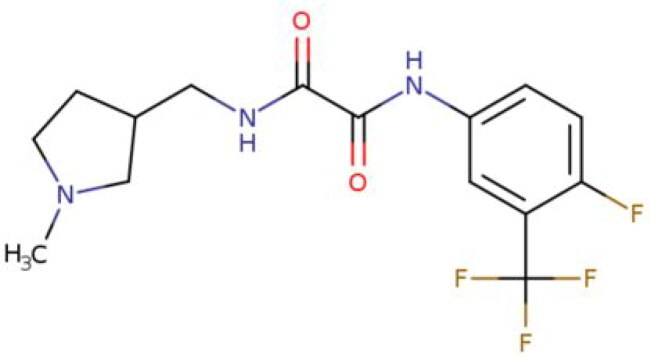	3	2	347.312	−13.733	−50.472	−52.74

* HBA = Hydrogen Bond Acceptor.

* HBD = Hydrogen Bond Donors.

† Molecule codes were repeated as there are the different conformers of the same molecules.

**Table 3.** Hydrogen bonding and PI interactions of the 15 compounds that formed 18 unique conformers.

Sr. No	Enamine Z-Number/ PubChem ID	H-Bonding	Pi-pi Interactions	Pi-Cat Interactions
1	95203364	E368, E328, R220	W490, W524	W448, W490, W524, W424
2	19591105	E526, E328, W483	W483, W490	W524
3	48957080	W490, Y475, W424	W490	W448, W524, W424
4	56783035	E328, Y475, Y448	W490	W448, W524, W424
5^*†*^	53498488	V327, E328		W448, W524
6^*†*^	71867970	W483		W448, W424
7^*†*^	75432522	E368	W490	W448, W490, W424
8	797993	W490		W448, W424
9	97230286	E328	W483	W448, W490, W524
10	56788228	E328		W448, W424
11	75382816	E328		W448, W490, W524
12	25271794	W483	W524	W448, W524, R220
13	75431867	E368	W490	W448, W490, W424
14	53590748	V327, W483		W448, W424
15	72007806	W490		W448, W424

† Different conformers of the same molecule show the same interatomic interactions.

The top five complexes identified by MD simulations in the later sections were highlighted in red.

### 3.1. Reference docking experiments

**Figure 3.**
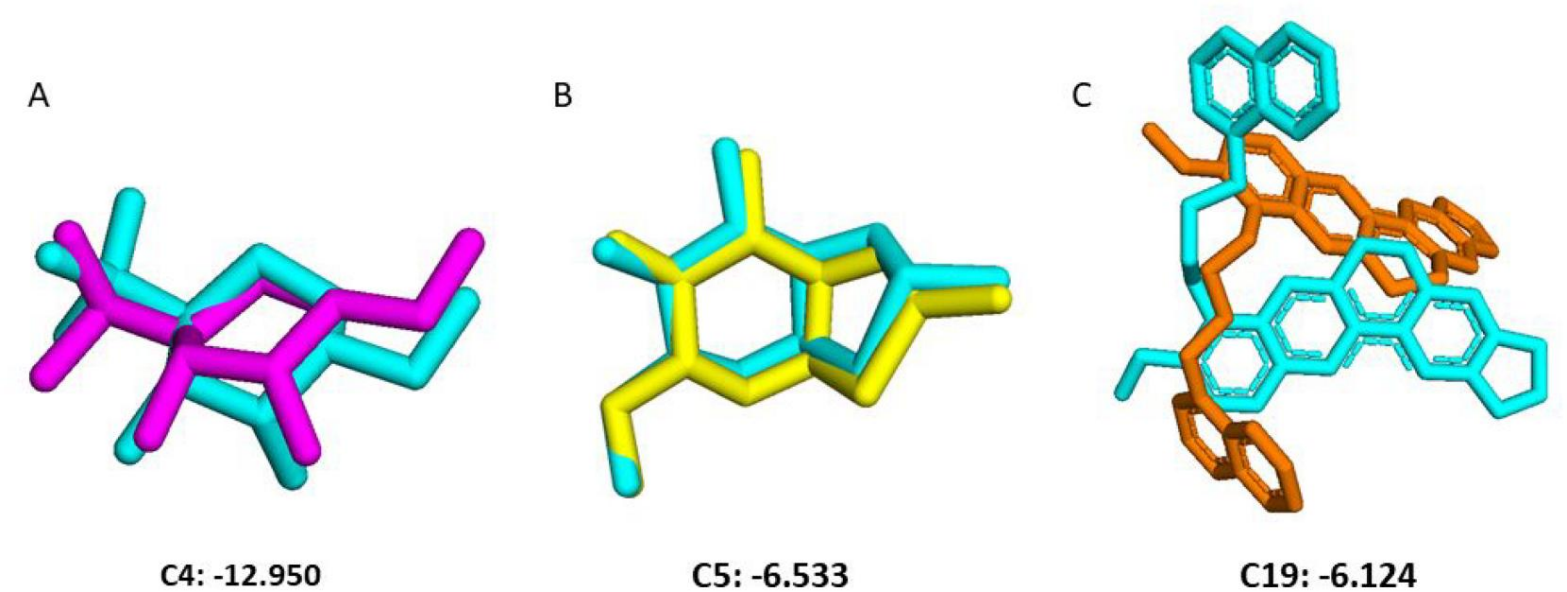
Superimposition of best docking poses on the co-crystallized ligands with their respective atom numbers and docking scores. The atom numbers represent the atom selected for receptor grid generation on Schrödinger Maestro interface. (A) Violet: Best docked pose of OfHex1 superimposed on the co-crystallized ligand (TMG-chitotriomycin) coloured in cyan (B) Yellow: Best docked pose of NAG-thiazoline in β-N-acetyl-D-hexosaminidase of *H. sapiens* superimposed on the co-crystallized NAG-thiazoline in cyan (C) Orange: Best binding pose of 8KL Berberine derivative in *T. pretiosum* Alphafold model of β-N-acetyl-D-hexosaminidase superimposed with that of the same derivative in the template (cyan).

In molecular docking studies, reference docking is of paramount importance in predicting the correct binding modes of ligands within protein binding sites, by evaluating the accuracy and reliability of docking algorithms (Korb et al., 2009). Reference molecular dockings of native ligands with the OfHex1 (PDB Id: 3NSN), *H. sapiens* (PDB Id: 1NP0), and *T. pretiosum* (Alphafold Model) have been performed before proceeding to virtual screening to assess the performance of the Glide docking. The co-crystallized ligands (TMG-chitotriomycin) and (NAG-thiazoline) in OfHex1 and *H. sapiens* respectively were first removed from the protein structure and then sketched, minimized, and redocked at the binding site. Redocking the native ligand provides a benchmark for assessing the algorithm’s ability to correctly predict the ligand’s binding mode and binding energy (Warren et al., 2006). For the *T. pretiosum* model, the native ligand from its template (5Y1B) was docked. The binding mode of the native ligand is usually experimentally determined, and previous studies shows that redocking enables researchers to see how well the docking algorithm can reproduce the experimentally observed binding mode (Muegge & Martin, 1999) (R. Wang et al., 2003). Best binding modes, with the glide scores of −12.950 kcal/mol, −6.533 kcal/mol and −6.124 kcal/mol for *O. furnacalis, H. sapiens,* and *T. pretiosum* respectively were superimposed on the co-crystal ligand as shown in Figure 3. For docking experiments of all three targets, receptor grids were generated based on the same coordinates’ information of the best poses shown in Figure 3.

### 3.2. Interatomic interactions of the native ligands with corresponding protein structures of β-N-acetyl-D-hexosaminidase in O. furnacalis, H. sapiens and the model of T. pretiosum

Interatomic interactions for the native ligands and their corresponding proteins, TMG-chitotriomycin in OfHex1, NAG-thiazoline in β-N-acetyl-D-hexosaminidase of *H. sapiens* and 8KL Berberine derivative of the *T. pretiosum’s* β-N-acetyl-D-hexosaminidase model were mapped using Arpeggio.

**Figure 4.**
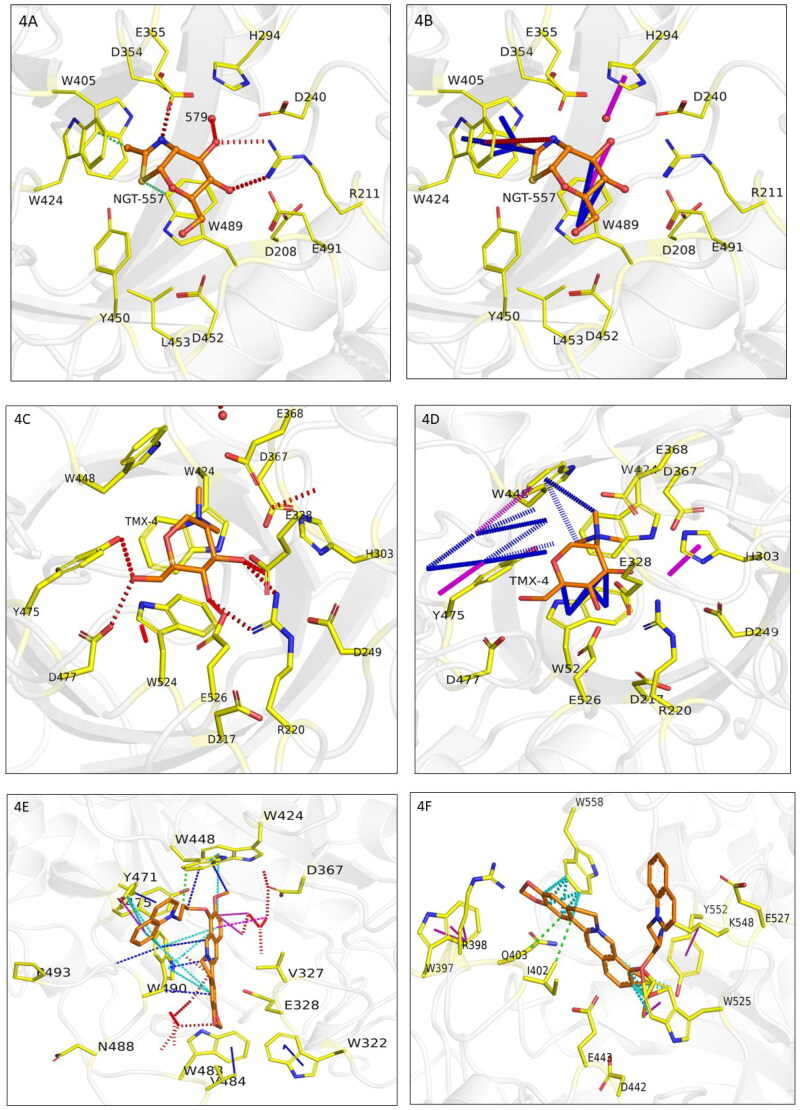
**A and B.** Interactions of NGT with 1NP0 (β-N-acetyl-D-hexosaminidase of *H. sapiens).* in 4 A hydrogen bonds are depicted in red and hydrophobic interactions in green. In 4B Carbon Pi interactions are shown in blue and Donor PI interactions in Magenta. **C and D.** Interactions of TMX with 3NSN (OfHex1*).* in 4 C hydrogen bonds are depicted in red. In 4D Carbon Pi interactions are shown in blue and Donor PI interactions in Magenta. **E and F.** Interactions of the berberine derivative (8KL) with the template 5Y1B (OfHex1 complexed with a berberine derivative) and Alphafold model of *T. pretiosum*. In 4E, binding site interactions of berberine derivative (8KL) with the template 5Y1B (OfHex1 complexed with a berberine derivative) are depicted. In 4 F, binding site interactions of Alphafold model of *T. pretiosum* β-N-acetyl-D-hexosaminidase complexed with the 8KL Berberine derivative are depicted. Hydrogen bonds: Red hydrophobic contacts: Green Aromatic contacts: Cyan; Carbon PI interactions in Magenta and Donor PI interactions in blue.

In the case of β-N-acetyl-D-hexosaminidase of *H. sapiens* (PDB ID: INP0) complexed with NGT (Figure 4A and B), residues W424, W489 and W405 indicate π–π interactions with the ligand while making a hydrogen bond with water molecule 579. In the case of *O. furnacalis* (PDB Id: 3NSN) complexed with (TMG-chitotriomycin), W448 and W524 are involved in making π–π interactions (Figure 4C and D). In *T. pretiosum’s* template (5Y1B), W448 and W490 were involved in making hydrophobic and π–π interactions with the Berberine derivative (8KL) while in the model of *T. pretiosum’s* β-N-acetyl-D-hexosaminidase, residues Q403, I402, W558, and Y552 were involved in making hydrophobic and π–π interactions with the template’s ligand (Berberine derivative (8KL)) (Figure 4E and F). Binding site residues are conserved across the three sites as noted by the interaction diagrams. Interestingly, a previous study also reveals a plus point that most of the catalytic center residues of β-N-acetyl-D-hexosaminidase of some harmful insects, like *Spodoptera frugiperda, Manduca sexta, Bombyx mori and Drosophila melanogester,* are conserved, as in *O.furnacalis.* Nevertheless, there exist various characteristics that can distinguish the enzymes from one another (Yang et al., 2008).

### 3.3. Identification of unique insecticide hits against OfHex1 through virtual screening

In the realm of discovering inhibitors which are characterized by novel chemical scaffolds, virtual screening stands as a proficient strategy. In the present study, 44,943 conformers generated by LigPrep from the developed insecticides/chemical scaffold library were screened using the virtual screening workflow module on the Schrodinger suite. The Glide docking program in Maestro (Schrödinger Suite 2022-2) was used to identify 18 unique conformers of 15 compounds that docked only with OfHex1 (but not with the β-N-acetyl-D-hexosaminidase of *H. sapiens* or the Alphafold model of *T. pretiosum*) with docking scores ≤ −12.950 (energy changes less than the reference ligand (TMG- chitotriomycin)) were selected. These conformers were regarded as the hit set and the docking scores, binding energy, and molecular properties of this hit set (15 compounds) are described in Table 2.

### 3.4. Protein-ligand interactions of all the 18 unique conformers against OfHex1

Table 2 depicts Glide binding energy of ≤ −31 kcal/mol and Glide G-scores of ≤ −12.96 for all the *O. furnacalis* unique 18 compounds indicating that these molecules have good binding affinities with OfHex1 compared to the reference molecule. Notably, all the compounds have N + H+ atoms in their structures. Upon docking analysis, most of the compounds show an orientation of terminal aromatic rings towards the pocket center depicting related binding patterns in the OfHex1 binding pocket. In a previous study of OfHex1, W448 and W490 were confirmed to be the vital residues for the binding of TMG-chitotriomycin inhibitor and their mutation reduced the activity of the enzyme by 2000-fold (T. Liu et al., 2011). The three tryptophan residues (W424, W448, and W524) form a hydrophobic core of the OfHex1 binding pocket and are highly conserved. The conserved catalytic triad of (D249, H303, and E368) and a water molecule H_2_O(II) at the end of the structure of this triad are stabilized by hydrogen bonding (T. Liu et al., 2011). Our reference docking approach also confirms that another water molecule, H_2_O(I), functions to stabilize E328 and E368. The crystal structure of OfHex1 revealed that all these catalytic residues are highly conserved.

One reason for choosing Ofhex1 in this study is that there are two Ofhex1-specific conserved loops (L314–335 and L478– 496) that are non-existent in human β -N-acetyl-D-hexosaminidases. Moreover, it is important to mention that V327 and W490 residues of Ofhex1 are present within these conserved loops because they are present at the opening of the binding pocket and can serve as a scaffold to stabilize the +1 sugar of the N acetyl Glucosamine substrate (Dong et al., 2019). Currently, although the binding mode of OfHex1 with its inhibitors remains unclear, we hypothesize that all these residues might be involved in the interactions with inhibitors. Therefore, the analysis of all the docking conformations of top hits has been done and only those ligands which interact with these key residues by π–π interactions or hydrogen-bonding interactions and which also possess high binding scores (Glide g-score < −12.950) and low binding energy (glide bind energy ≤ −31 kcal/mol) were kept for further analysis. Keeping in mind, all the important OfHex1 residues and the results of reference docking, 5 compounds from the total 18 hits with the π–π or hydrogen-bonding interactions with residues W490, W483, E328, E368, and E526 were obtained for MD simulations. All the Ofhex1 specific 18 conformers (of 15 compounds) and their docked interactions are described in Table 3.

### 3.5. Molecular dynamics simulation

In order to determine the consistency of docking and the interatomic interactions between the target proteins and the screened ligands, all the docked complexes of 18 conformers for OfHex1 were initially subjected to 5 ns MD simulations on the Desmond Suite. The 5 ns simulation was done as an initial assessment of the system to ensure its stability and equilibration. Only 5 compounds from among the 18 conformers showed protein and ligand RMSD varying across the trajectory with an average less than 3 Å. Hence, these five compounds with Pubchem IDs 19591105, 95203364, 48957080, 53498488, 56783035 were subjected to a 40 ns MD simulation to check their stabilities over a long-range simulation in the OfHex1 binding pocket. Previous studies revealed that running a short simulation before a long-range simulation validates the simulation protocol, force field, solvent model and other simulation settings and is helpful in saving time and resources (Braun et al., 2019). The RMSD values were raised at the beginning, indicating that the complexes are removing repulsions and relaxing in the solvent systems. Compound 95203364 gained the equilibrium state at 5 ns and remained at equilibrium till the end, with a Cα RMSD value of around 2 Å, Compound 48957080 and Compound 19591105 reached the stable equilibrium after 25 ns with an RMSD value of around 2.5 Å.

**Figure 5.**
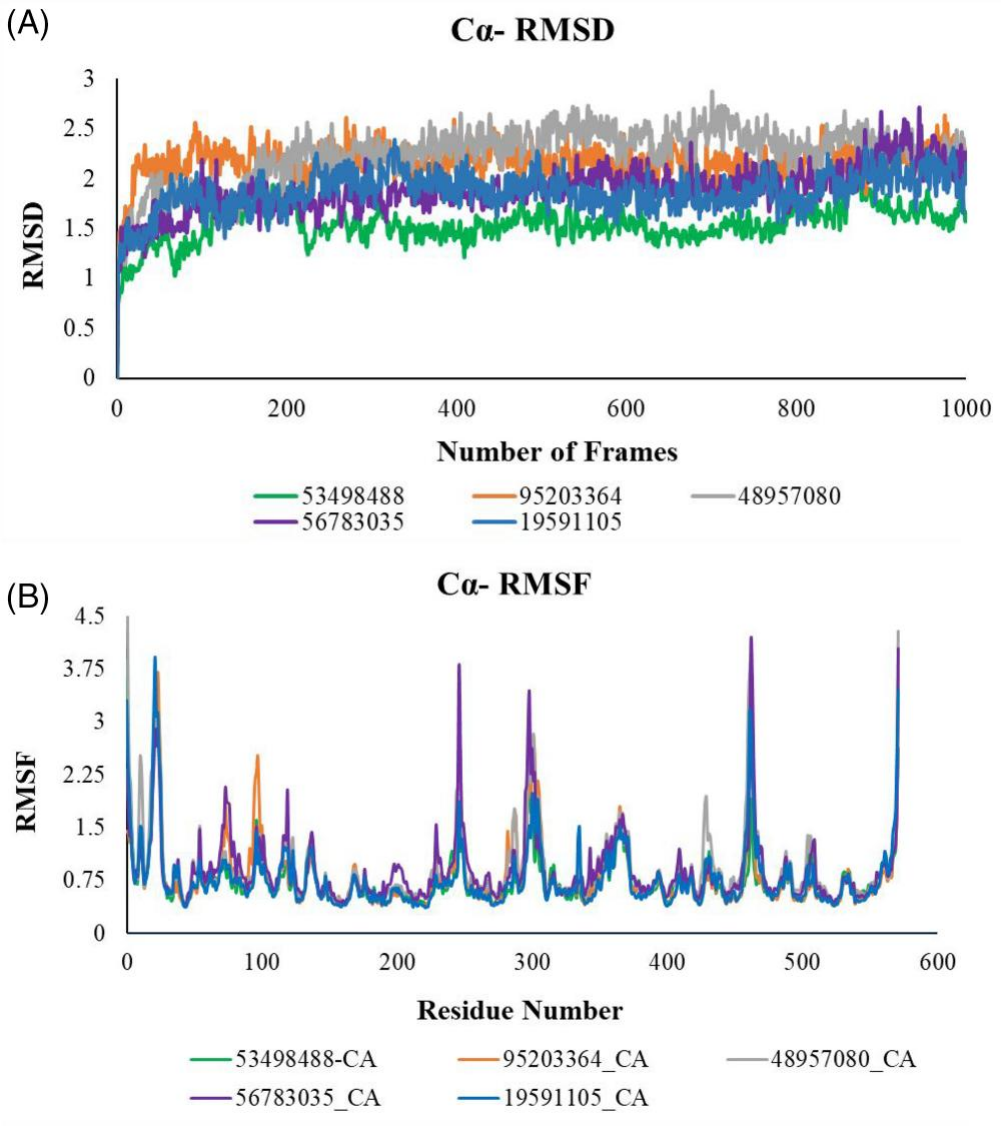
**A**. Root mean square deviations (RMSD) of all Cα atoms of the complexes during the production phases relative to the initial structures. (green, 53498488; Orange, 95203364; grey, 48957080; purple, 56783035; blue, 19591105). **B**. Root mean square fluctuations (RMSF) of an individual residue of ofhex1 for top five compounds identified in MD simulation. (Green, 53498488; Orange, 95203364; grey, 48957080; light purple, 56783035; blue, 19591105).

As noted in Figure 5A, all the five complexes fluctuated around a thermal average structure with an Cα-RMSD ranging from 0.5 Å to 2.5 Å indicating that the complexes are stable and there are no large-scale conformational changes throughout the course of the trajectory.

In Figure 5B, we note that fluctuations in residues occur around the residues numbered between 220 and 350. This could be due to the hydrogen bond interactions that arginines and glutamates make with the ligands in the complexes in this residue range (please see Table 3). Similarly, we also noted large scale fluctuations in the range of 430-500 amino acids residues. This could be attributed to the network of donor-pi, carbon-pi and cation-pi interactions that the tryptophans made in residue range.

### 3.6. Interatomic interactions of the top five hits

**Figure 6.**
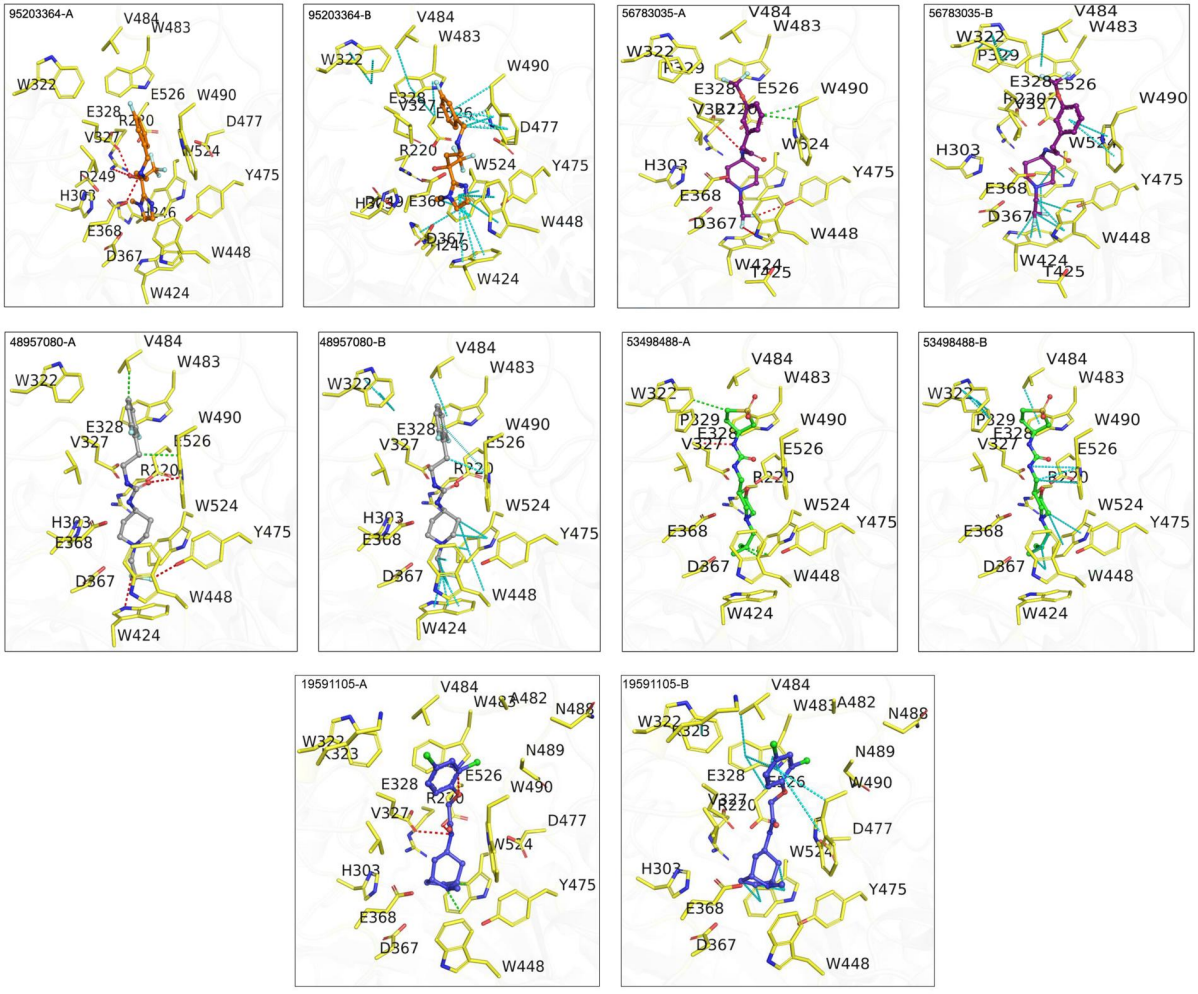
Protein-Ligand Interactions of top five compounds that docked in the active site of OfHex1. (A) Hydrogen bonds colored in red and hydrophobic contacts in green (B) Aromatic contacts colored in cyan.

From the long-range MD simulations, we analyzed the consistency of some of the contacts that some of the residues make with the ligands in the complexes. We noted that for the compound 95203364, W490 and W524 formed pi-interactions with the ligand that remained 60% and 95% consistent throughout the simulation trajectory. E368 formed multiple hydrogen bonds, ionic and water bridges with the ligand. For the compound 19591105, W424 and W448 formed pi-interactions which are 90% consistent through the simulation. Additionally, W475 formed critical hydrogen bond interactions that remained nearly 100% consistent throughout the trajectory. For compound 48957080, we noted ionic and water bridge interactions of E368 and pi interactions of W524 that remained consistent for 100% and 85% of the trajectory respectively. For the compound 56783035, we noted E368 forming hydrogen bond, ionic and water bridge interactions consistently throughout the trajectory. Residues W475 and W490 also formed hydrogen bond and pi-interactions with 100% and 65% consistency respectively. For the compound 53498488, W524 and E526 formed consistent pi and hydrogen bond interactions (Supplementary Data 2 and Figure 6).

### 3.7. Characterization of insecticide/pesticide likeliness

Qualitative and quantitative assessment of pesticide likeness based on the properties of molecules was done by Comprehensive Pesticide Likeness Analysis (CoPLA). The qualitative evaluation methods consider the physicochemical properties of compounds, toxicity characteristics, lipophilic profile, water solubility, photostability, permeability, flexibility, molar refractivity, and topological structure etc., while quantitative assessments are based on relative drug likelihood (RDL), quantitative estimate of pesticide likeness (QEP) scores and gaussian scoring function (GAU). The QEP scores range from 0 to 1, which can be calculated by considering exponential desirability functions of various descriptors and then executing conditional summations. The range of RDL score is wide and a value >1 represents a higher pesticide likeness, whereas a value <1 specifies a low pesticide likeness (Zhao et al., 2022; Avram et al., 2014).

CoPLA does pesticide-likeness property assessment based on the Tice Rule for insecticides, Tice Rule for herbicides and Hao Rule for pesticides (M. Wang et al., 2019). Just like Lipinski’s rule of five (Ro5) for optimal bioavailability of drug candidates, Tice’s rule of five was implemented to profile the different types of agrochemicals. Keeping in mind the molecular descriptors for pesticide likeness, the Tice rule considers molecular weight (MW) ≥ 150 and ≤ 500, atomic logP (AlogP) ≥ 0 and ≤ 6.5, hydrogen bond acceptors (HBA) ≥ 1 and ≤ 8, hydrogen bond donors (HBD) ≤ 2 and the number of rotatable bonds (RB) ≤ 12 while Hao rule for pesticides also considers the number of aromatic bonds (aRr) along with all the five rules (Tice, 2001). In order to overcome the limitations of traditional qualitative methods, three quantitative estimation methods of pesticide likeness were established. QEP scoring function estimates the pesticide likeness based on the already present pesticides considering the RO5. RDL function is based on the Bayesian probability theory and evaluates the distributions of differences of physical and chemical properties between the pesticidal and non-pesticidal compounds. The third function is the Gaussian function which is different from others in the sense that it gives information on more multivariate descriptors of molecules including the RO5 along with the polar surface area (PSA), number of nitrogen atoms (nN), number of oxygen atoms (nO) and number of rings (nRings) (Jia et al., 2019).

All five compounds show low-risk toxicity probability in both humans and honeybees. Compounds 56783035, and 48957080 are accepted under all three rules: Hao Rule for pesticides, Tice Rule for herbicides, and Tice Rule for insecticides, compound 19591105 shows acceptance under Hao Rule for pesticides and Tice Rule for herbicides while compound 53498488 and compound 95203364 shows acceptance under Tice Rule for herbicides b giving the alert, i.e. AlogP values ≥ 0 and ≤ 6.5 where AlogP estimates are only suited to compounds having C, H, O, N, S, Se, P, B, Si, and halogens, no complex aromaticity and electronic systems that contribute to logP. Compounds 95203364 and 53498488 are predicted to exhibit marginally lower AlogP values of −1.181 and −1.5709, than the desired 0, respectively. The slight variation might be associated to relatively higher inherent electron resonance as exhibited by both the ligand chemical structures, which is though very much negligible. Given the fulfillment of rest of all CoPLA characteristics as mentioned in Table 4, this minute variation might be ignored. In a previous study by Ghose and Crippen, a similar method to Lipinski’s RO5 was considered and alogP method was developed to calculate logarithm of the octanol/water partition coefficient (Ghose & Crippen, 1987). The qualifying ranges for that method include MW = 160-480, total number of atoms = 20-70, molar refractivity = 40-130 and alogP = −0.4 − 5.6 (Jia et al., 2019; Ghose & Crippen, 1987). Agrochemicals are delivered most frequently by spraying and the main difficulty faced by agricultural chemists while spraying is finding the compounds that selectively bind to the right receptor in target species and have the right physicochemical properties to reach that target. Pesticides need to cross varied barriers to be absorbed and are used more occasionally than pharmaceutical doses. However, for maximizing penetration, pesticide formulation can be modified (Tice, 2001).

**Table 4.** Comprehensive Pesticide likeness analysis (CoPLA) of the top five compounds identified in MD simulation.

Sr No.	Molecule IDs	ALogP	Mw	HBA	HBD	RB	arR	GAU	QEI	RDL
1	19591105	−0.4844	342.09	3	1	5	1	5.46	0.59	1.39
2	95203364	−1.181	332.14	3	3	7	0	5.24	0.35	1.49
3	48957080	0.0499	347.16	4	2	7	1	5.18	0.59	1.24
4	53498488	−1.5709	333.17	7	2	6	0	4.48	0.20	1.13
5	56783035	0.9565	348.14	3	1	8	1	6.07	0.77	1.35

* ALogP (log of the octanol-water partition coefficient), Mw (molecular weight), HBA (number of hydrogen bond acceptors), HBD (number of hydrogen bond donors), RB (rotatable bonds), arR (number of aromatic rings), GAU (Gaussian scoring function), QEI (quantitative estimate of insecticide likeness), RDL (relative drug likelihood).

## 4. Conclusion

*O. furnacalis* β-N-acetyl-D-hexosaminidase (OfHex1) is an essential enzyme involved in insect chitin catabolism and an appealing target for the development of eco-friendly insecticides. To the best of our understanding, this study marks the first *in-silico* investigation through the utilization of already-developed insecticides or core agrochemical scaffolds compounds library to discover OfHex1 specific inhibitors. Furthermore, our study encompassed virtual ligand screening, binding free energy calculations, and molecular dynamic simulations to explore the potential mechanisms of binding with OfHex1 inhibitors. We identified 18 unique conformer hits (from 15 compounds) specifically against OfHex1 which do not bind with Human HEX and *T. pretiosum* HEX. Notably, five of these compounds demonstrated consistent binding in the TMX binding site (active site). Our 40 ns molecular dynamics analysis highlighted the significance of π–π stacking interactions with W490 and W524, as well as intermolecular hydrogen bonding with E328 and Y475, in stabilizing ligands within the receptor. The results obtained through MM-GBSA calculations further substantiated that the five potential inhibitors exhibited favorable binding affinities with OfHex1. All these five compounds with Pubchem IDs 19591105, 95203364, 48957080, 53498488, 56783035 represent a diverse class of chemical scaffolds and show low-risk toxicity probability in both humans and honeybees upon CoPLA analysis. Hence, we believe that these compounds could be useful for the ongoing development of more potent and novel OfHex1 inhibitors. However, further *in-vitro* validations are required to demonstrate their utility and importance as potential insecticide-like compounds against β-N-acetyl-D-hexosaminidase in *O. furnacalis*.

## Supplementary Material

Supplemental Material
