# Supplementary material for: Structure-guided computational insecticide discovery targeting β-N-acetyl-D-hexosaminidase of Ostrinia furnacalis
Source: J Biomol Struct Dyn. 2023 Oct 9;42(21):11717–30. doi: 10.1080/07391102.2023.2264394 (PMC11573315; doi:10.1080/07391102.2023.2264394)
Supplement: Supplemental Material [file TBSD_A_2264394_SM4505.zip › suppl_data/Supplementary Data 1.pdf]

Supplementary Data

Supplementary Table 1

All XP Docked compounds with *Ostrinia furnacalis* β-N-acetyl-D-hexosaminidase (3NSN)

Sr No.	Pubchem ID/Z ID	Formula	glide_XP_GScore	MMGBSA_dG_Bind
1	Z1517973754	C15H20F4N2O	-16.8524	-50.9332
2	Z1518004134	C15H20F4N2O	-17.1402	-40.7346
3	Z1518004134	C15H20F4N2O	-16.1783	-31.6984
4*	Z1515085646†	C15H21F4N3	-15.9358	-43.7256
5*	Z1508324634	C15H21ClF3N3	-15.8759	-44.7382
6	Z1439564204	C18H29N3OS	-15.0372	-39.4247
7	Z1439564204	C18H29N3OS	-14.9317	-44.7048
8	Z1637133799	C16H30N2S	-14.3163	-54.3375
9	Z1529442542	C16H32N4OS	-14.4193	-41.5994
10	Z902906564	C14H17F4N3O	-14.2368	-37.8295
11	Z1517973754	C15H20F4N2O	-14.2274	-46.3288
12	Z902906564	C14H17F4N3O	-14.1304	-38.0945
13*	Z1184006932	C16H21F4N3O	-14.4947	-28.2501
14*	Z1237763936	C15H17F4N3O2	-13.7331	-52.7389
15*	Z1307575992†	C17H34N4OS	-14.0285	-53.2106
16*	Z1148360224	C16H20F4N2O2	-14.8338	-24.1031
17	Z1637133799	C16H30N2S	-13.5822	-50.067
18	Z1439564204	C18H29N3OS	-13.7554	-35.9784
19*	Z1515085646	C15H21F4N3	-13.8598	-50.5142
20*	Z951575534	C16H21F4N3O	-13.9211	-29.7326
21*	Z875436476	C15H19F4N3O	-13.4155	-28.7855
22	Z1569561365	C15H18F4N2O	-13.3306	-7.90299
23*	Z1437933420	C16H22F4N2	-14.5487	-44.3103
24	Z1518004134	C15H20F4N2O	-13.6902	-47.5882
25	Z1702861401	C13H8ClF3N2O4	-13.1558	-32.5017
26	Z951565562	C16H21F4N3O	-13.6874	-41.1777
27*	Z432456704†	C14H27N3O4S	-13.2103	-17.9832
28*	25271794	C13H22N5O2	-13.1064	-52.0256
29*	Z1307575992	C17H34N4OS	-13.4163	-38.7151
30*	Z432456704	C14H27N3O4S	-13.1344	-38.1872
31*	Z1079048558	C15H17F4N3O	-13.6799	-68.8054
32	Z1141301921	C14H26N2S	-13.2432	-37.0769
33*	19591105	C16H20Cl2N2O2	-12.9597	-41.5487
34	Z974419392	C15H18F4N2O	-12.9209	-48.275
35	Z1637133799	C16H30N2S	-12.8844	-51.3651
36*	Z1437933218	C15H18F6N2	-14.2193	-45.5633
37	Z1079048648	C15H16F5N3O	-13.5156	-73.7994
38	Z1518004134	C15H20F4N2O	-14.0786	-31.732
39	Z1608885533	C19H31N3S	-12.8216	-38.547

40	Z1497795855	C15H20N2OS2	-12.7461	-23.9311
41*	797993	C14H20N2O	-13.2062	-32.5888
42	Z1497795855	C15H20N2OS2	-12.6996	-28.4354
43	Z952060658	C19H26N2OS	-12.7331	-35.2738
44	Z1608885533	C19H31N3S	-12.697	-42.4222

† Molecule codes were repeated as there are the different conformers of the same molecule.

**18 Molecules that show binding only with Ostrinia furnacalis but not with Tricogramma pretiosum and Homosapiens.*

Supplementary Table 2

All XP Docked compounds with *Tricogramma pretiosum* β-N-acetyl-D-hexosaminidase (Alphafold Model)

Sr No.	Pubchem ID/Z ID	Formula	glide_XP_GScore	MMGBSA_dG_Bind
1	Z1517973754	C15H20F4N2O	-12.5665	-20.4071
2	Z1268663450	C16H17F4N3	-12.6706	-20.108
3	Z1490864225	C14H19ClF4N2O	-12.7627	-25.059
4	Z1518004134	C15H20F4N2O	-12.0926	-14.635
5	Z1518004134	C15H20F4N2O	-11.934	-7.91428
6	Z1440466968	C18H27N3OS	-11.507	-31.3341
7	Z1440466968	C18H27N3OS	-11.3407	-35.6311
8	Z1440466968	C18H27N3OS	-11.1173	-25.6476
9	Z1440466968	C18H27N3OS	-11.108	-27.4625
10	Z1518004134	C15H20F4N2O	-11.4201	-24.7952
11	Z1490864225	C14H19ClF4N2O	-11.9679	-22.4667
12	20056441	C22H32N5O19P	-12.0652	-25.0454
13	20056441	C22H32N5O19P	-11.0109	-25.5777
14	Z1440466968	C18H27N3OS	-10.7593	-31.3098
15	Z1569561365	C15H18F4N2O	-10.5065	-14.4623
16	Z1689270476	C15H18F4N2O2	-10.5495	-3.12869
17	Z1517926282	C16H17F4N3	-10.9143	-27.3476
18	20056441	C22H32N5O19P	-10.7151	-23.7797
19	Z1440466968	C18H27N3OS	-10.4955	-26.7713
20	Z1561886630	C13H14ClF3N2O	-10.3766	-16.3068
21	Z31168692	C12H6ClF3N2O4	-10.2518	-18.7628
22	Z1490864225	C14H19ClF4N2O	-11.2089	-10.1917
23	Z1689270476	C15H18F4N2O2	-10.2448	-7.49823
24	Z107133200	C14H10ClF3N2O3	-10.1615	-30.5388
25	Z18546791	C13H7F5N2O2S	-9.97051	-24.8248
26	20056441	C22H32N5O19P	-10.1192	-15.5219
27	Z1690056454	C11H18F6N2O2	-9.86126	-12.4839
28	145050221	C9H9FN3S	-9.73886	-18.9421
29	Z1690056454	C11H18F6N2O2	-9.58035	-13.6704
30	20056441	C22H32N5O19P	-9.8156	-31.3652

31	Z1690123507	C15H20F4N2O	-9.47795	-17.6832
32	Z991982568	C14H25N3O3S	-9.43575	-23.6738
33	Z222999194	C13H16ClF3N4O	-9.88702	-24.9519
34	Z1587916204	C12H13F6NO	-9.59867	-15.6132
35	Z1587916204	C12H13F6NO	-9.58626	-6.55087
36	Z1517973754	C15H20F4N2O	-9.40164	-30.6836
37	Z1317985800	C15H18F4N2O2	-9.42207	-16.0445
38	Z902906564	C14H17F4N3O	-9.37296	-25.5556
39	20056441	C22H32N5O19P	-9.61605	0.6583
40	Z1689269260	C14H16F4N2O2	-9.56129	-6.54718
41	20056441	C22H32N5O19P	-9.51131	-29.8578
42	Z1689261929	C10H16F6N2O2	-9.3077	-2.26037
43	Z1831626363	C12H20F6N2O2	-9.66774	6.545464
44	Z1206306181	C10H17F4N3O	-9.25186	-6.2871

Supplementary Table 3

All XP Docked compounds with *Homo sapiens* β-N-acetyl-D-hexosaminidase (1np0)

Sr No.	Pubchem ID/Z ID	Formula	glide_XP_GScore	MMGBSA_dG_Bind
1	Z1417107779	C19H31N3OS	-14.205	-33.0955
2	Z1563054593	C19H31N3S	-14.2474	-17.8892
3	Z1439564204	C18H29N3OS	-14.0348	-46.9159
4	426840	C12H18Cl2NO2	-13.6141	-35.0611
5	Z1638891435	C18H28N2OS	-13.4169	-56.3145
6	44973382	C18H24ClN3O5S	-13.4836	-31.042
7	Z1439564204	C18H29N3OS	-13.4547	-46.8884
8	Z1417107779	C19H31N3OS	-13.0693	-35.6324
9	Z1637133799	C16H30N2S	-13.0631	-36.9405
10	Z1638891435	C18H28N2OS	-13.0305	-41.3862
11	Z506420796	C15H27N3O3S	-12.9892	-27.0157
12	Z1518846165	C17H25N3OS	-12.8999	-38.6752
13	Z750912840	C13H25N3O3S	-12.8409	-33.3582
14	Z506420796	C15H27N3O3S	-12.7857	-39.1738
15	Z1563054593	C19H31N3S	-12.7912	-26.5857
16	Z1529442542	C16H32N4OS	-12.7387	-25.3737
17	Z951565562	C16H21F4N3O	-13.095	-32.5437
18	Z168462736	C17H24N2O3S	-12.5614	-29.1169
19	Z1517973754	C15H20F4N2O	-12.4668	-34.9641
20	Z1637133799	C16H30N2S	-12.4437	-44.2057
21	Z1420494122	C17H19F4N3	-13.8023	-27.7659
22	Z31168692	C12H6ClF3N2O4	-12.3805	-6.68736
23	Z510737672	C13H25N3O3S	-12.3179	-13.6408
24	Z991982568	C14H25N3O3S	-12.2994	-17.9257
25	Z1440398142	C18H29N3S	-12.397	-32.1657
26	Z1079048648	C15H16F5N3O	-12.9409	-65.0587

27	Z1702861401	C13H8ClF3N2O4	-12.2614	4.611364
28	Z1440398142	C18H29N3S	-12.3191	-35.3513
29	Z1426132857	C16H27N5S	-12.3908	-31.5938
30		$C14H30N2^{2+}$	-12.1332	-27.5401
31	Z437495108	C13H25N3O4S	-12.1277	-15.8058
32	Z1141301921	C14H26N2S	-12.3558	-31.0442
33	Z1306478211	C16H31N3OS	-12.023	-33.0056
34	Z532936096	C13H25N3O3S	-12.0096	-33.3002
35	Z951561828	C14H17F4N3O	-12.8289	-34.0715
36	Z167413474	C16H22N2O3S	-12.5936	-21.6369
37	135539635	C14H16BrN4O2	-11.9729	-41.2057
38	Z1283640390	C14H16F4N2O	-11.946	-34.7121
39	Z826945434	C14H15ClF3N3O2	-11.8846	-38.1936
40	Z46169079	C13H14ClF3N2O	-11.8508	-13.9896
41	Z1440703756	C18H35N3S	-11.9243	-32.2646
42	Z532936096	C13H25N3O3S	-11.7777	-19.5105
43	Z1139725211	C14H27N3O4S	-11.7734	-22.8205
44	Z1608885533	C19H31N3S	-11.8519	-26.3706
